# Non-Invasive Three-Dimensional Cell Analysis in Bioinks by Raman Imaging

**DOI:** 10.1021/acsami.1c24463

**Published:** 2022-07-01

**Authors:** Julia Marzi, Ellena Fuhrmann, Eva Brauchle, Verena Singer, Jessica Pfannstiel, Isabelle Schmidt, Hanna Hartmann

**Affiliations:** † NMI Natural and Medical Sciences Institute at the University of Tübingen, Reutlingen 72770, Germany; ‡ Institute of Biomedical Engineering, Department for Medical Technologies & Regenerative Medicine, Eberhard Karls University, Tübingen 72074, Germany; § Cluster of Excellence iFIT (EXC 2180) Image-Guided and Functionally Instructed Tumor Therapies, University of Tübingen, Tübingen 72074, Germany

**Keywords:** bioinks, extrusion-based bioprinting, non-invasive cell analysis, Raman microspectroscopy, molecular imaging

## Abstract

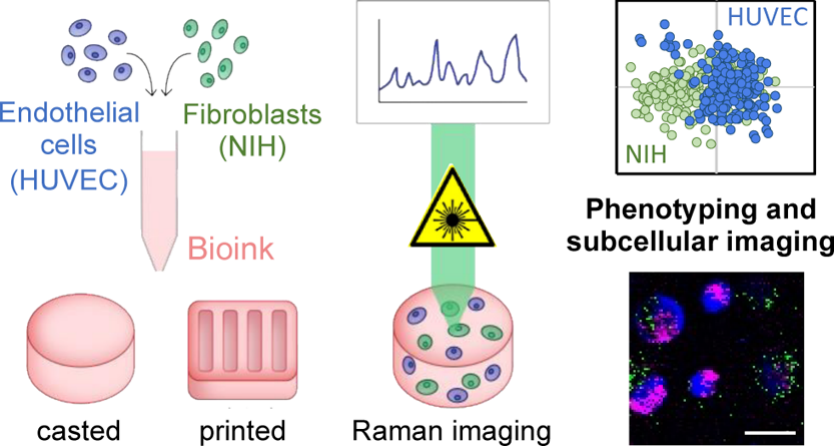

3D bioprinting is an emerging biofabrication strategy using bioinks, comprising cells and biocompatible materials, to produce functional tissue models. Despite progress in building increasingly complex objects, biological analyses in printed constructs remain challenging. Especially, methods that allow non-invasive and non-destructive evaluation of embedded cells are largely missing. Here, we implemented Raman imaging for molecular-sensitive investigations on bioprinted objects. Different aspects such as culture formats (2D, 3D-cast, and 3D-printed), cell types (endothelial cells and fibroblasts), and the selection of the biopolymer (alginate, alginate/nanofibrillated cellulose, alginate/gelatin) were considered and evaluated. Raman imaging allowed for marker-independent identification and localization of subcellular components against the surrounding biomaterial background. Furthermore, single-cell analysis of spectral signatures, performed by multivariate analysis, demonstrated discrimination between endothelial cells and fibroblasts and identified cellular features influenced by the bioprinting process. In summary, Raman imaging was successfully established to analyze cells in 3D culture in situ and evaluate them with regard to the localization of different cell types and their molecular phenotype as a valuable tool for quality control of bioprinted objects.

## 1 Introduction

Using additive manufacturing techniques such as three-dimensional (3D) bioprinting, tissue-like structures for regenerative medicine and functional in vitro models can be realized in a layer-by-layer fashion. In extrusion-based bioprinting, biological material is deposited in continuous filaments of bioink by applying pneumatic or mechanical forces. Fabrication of organotypic tissues requires the arrangement of different cell types in a defined manner. Hydrogels are often used to encapsulate cells in so-called bioinks during the bioprinting process to ensure cell survival and provide shape stability to the printed object.^1−3^ Hydrogels are formed from synthetic or natural network polymers with the ability to absorb large amounts of water or biological fluids.^4^

Natural hydrogels such as alginate are widely used in various bioprinting applications.^5^ Alginate is a naturally occurring anionic polysaccharide typically obtained from brown seaweed which shows similarities to polysaccharide structures of glycosaminoglycans found in the extracellular matrices of living tissues. Gelation of alginate is induced in the presence of divalent cations such as Ca^2+^. Alginate is biocompatible and has been widely used in the formulation of bioinks.^4,5^ However, alginate does not possess binding motifs that are recognized by cells, and thus, cell adhesion to alginate is limited.^4^ To overcome this limitation, other biomaterials such as gelatin are often used as additives in multimaterial inks. Gelatin, a denatured collagen derived from animal bones, tendons, or skins, is thermoresponsive and forms a hydrogel at lower temperatures. Moreover, gelatin contains intrinsic Arg–Gly–Asp (RGD) motifs that can mediate cell adhesion.^6^

Another limitation in the use of alginate solutions as bioinks is their low mechanical properties and shape fidelity.^7^ At low shear rates, alginate precursor solutions display generally low viscosity and Newtonian flow behavior.^8^ The viscosity of alginate can be increased by the polymer concentration and molecular weight in order to improve printability of alginate-based bioinks.^9^ While more viscous bioinks achieve good printability, cell viability is often compromised.^10^ These opposing material requirements have been conceptualized by Malda et al. and are known to the scientific community as the “biofabrication window”.^11^ One possibility to overcome this problem is the use of additives such as nanofibrillated cellulose (NFC) to increase the viscosity of alginate precursor solutions.^8^ NFC by itself has high viscosity at low shear rates and is shear thinning.^12^ Shear thinning behavior in extrusion printing is favorable since it allows a lower dispensing pressure during printing, which is beneficial for cell viability.^13^ By combining alginate and NFC, a bioink with the rheological properties of NFC and the cross-linking ability of alginate has been formulated.^12,14^

Biological evaluation is essential to analyze cell behavior within bioprinted objects. Recent studies often focus on cell viability using commercial assays such as calcein–AM and ethidium bromide or propidium iodide staining (live/dead assay), lactate dehydrogenase assay, trypan blue assay, and annexin V staining.^15^ Besides the lack of consideration of other cellular processes such as cell differentiation, these assays are destructive end point measurements. Since bioprinted objects for applications in regenerative medicine and functional in vitro models, for example, with patient-specific cells and tissues, can be regarded as unique objects, non-invasive methods to evaluate cells within bioinks are needed.

Here, we investigated the suitability of Raman microspectroscopy as an in situ monitoring tool for 3D and bioprinted objects. Raman spectroscopy has been employed as a non-invasive and non-destructive technique in numerous biomedical applications in 2D cell culture.^16^ Raman spectra of a biological specimen can provide subcellular information on the content of components such as proteins, nucleic acids, and lipids, allowing us to assess cell differentiation, phenotype, or cell death stages in situ.^17−19^ Thus, living cells and tissues can be identified based on their biochemical signature under physiological conditions. In addition to single-point microspectroscopy, Raman imaging has the potential to provide both molecular-sensitive characterization as well as spatial localization of cellular structures by the generation of hyperspectral maps. Conventional methods such as immunofluorescence staining, cytometry, or gene expression assays only provide limited access to this information. Therefore, we aimed to implement Raman imaging as a non-destructive in situ tool to assess cellular information in a 3D culture environment, which could thereby lead to new readouts, improving the analysis of bioprinted objects.

In this study, we investigated different bioinks by non-invasive Raman microspectroscopy and demonstrated the marker-independent discrimination of two exemplary cell types by multivariate data analysis. Spectral analysis allowed us to distinguish cell types in printed and cast 3D objects as well as to spatially resolve their major subcellular structures. In summary, we show that Raman imaging is a promising tool to analyze cells in 3D culture and evaluate bioprinted objects in a non-destructive manner.

## 2 Materials and Methods

### 2.1 Cells

Two different cell types of different physiological functions were selected as exemplary cell types for the following experiments. NIH-3T3 cells were obtained from ATCC, USA, and cultured in phenol red-free Dulbecco’s modified Eagle’s medium (Life Technologies, USA) supplemented with 10% FBS (Life Technologies, USA), 4 mM l-glutamine (Life Technologies, USA), 1 mM sodium pyruvate (Life Technologies, USA), and 50 U penicillin/streptomycin (Life Technologies, USA) in a humidified incubator at 37 °C and 5% CO_2_. Human umbilical vein endothelial cells (HUVEC) were obtained from PromoCell and cultured in phenol red-free endothelial cell basal medium (PromoCell, Germany) supplemented with a supplement mix (PromoCell, Germany) and 50 U penicillin/streptomycin (Life Technologies, USA) in a humidified incubator at 37 °C and 5% CO_2_.

### 2.2 Cell Morphology

The actin cytoskeleton and nuclei (DNA) of cells were fluorescently labeled for visualization in 2D and 3D. In 2D, 5000 cells/cm^2^ were seeded on tissue culture plates and cultured for 24 h prior to staining. In 3D, 1 × 10^6^ cells/mL were mixed into the bioinks and cultured for 24 h prior to staining. For staining in 2D, cells were fixed using 4% paraformaldehyde (PFA, Carl Roth, Germany) for 30 min. After permeabilization with 0.02% Triton X-100 (Carl Roth, Germany) for 15 min and blocking in 1% bovine serum albumin (BSA, GE Healthcare, USA) for 1 h, cells were stained with 1 U/mL of phalloidin oregon green (Life Technologies, USA) for 3 h. Afterward, DNA was stained by incubating 50 μg/mL of DAPI (Sigma-Aldrich, USA) for 10 min. After each step, cells were washed three times in Dulbecco’s phosphate buffered saline (DPBS, Life Technologies, USA). Image acquisition was performed on an inverted fluorescence microscope (Zeiss, Germany). The same procedure was adopted for cells in 3D, with the following modifications: cells were fixed for 40 min on ice and 20 min at room temperature. Permeabilization with 0.5% Triton X-100 was performed for 30 min. Cells were stained with 2 U/mL of phalloidin oregon green overnight. Afterward, DNA was stained by incubating with 2.2 μg/mL of propidium iodide (PI, Sigma-Aldrich, USA) for 20 min. After each step, cells were washed three times in DPBS for 10 min. Image acquisition was performed on a confocal spinning disc microscope (Zeiss, Germany).

### 2.3 Coating

To prevent floating of cast and printed bioinks during Raman analysis from the substrate, cell culture dishes (Ibidi # 81156) were coated with polyelectrolyte multilayers. To this end, dishes were incubated with polyethylenimin (PEI, Sigma) for 10 min. After PEI was removed, dishes were washed three times for 2 min in ultrapure water. Then, polystyrolsulfonate (PSS, Sigma) and polyallylamin hydrochloride (PAH, Alfa Aesar) were incubated alternately one after the other for 10 min. This procedure was repeated to produce two bilayers of PSS/PAH. After each step, dishes were washed three times for 2 min in ultrapure water. Dishes were left at room temperature until completely dry. The contact angle was measured with EasyDrop (Krüss, Germany). Floating of printed objects was probed by adding cell culture medium on cross-linked and non-cross-linked samples (Supporting Information Figure S1).

### 2.4 Inks

Commercially available alginate-based ink was obtained from CELLINK, Sweden. Sodium alginate (Protanal LF 10/60 FT) was obtained from FMC Biopolymers, USA. Alginate inks were prepared as described elsewhere (Lorson et al. 2020). Briefly, a sodium alginate solution (0.5%, w/v) was first prepared by dissolving the powder in ultrapure water for 24 h at 25 °C and 60 rpm. The solution was then sterile filtered through a 0.2 μm polyethersulfone membrane (Thermo Fisher Scientific, USA) under a clean bench. Filtered alginate solution was then frozen in liquid nitrogen and lyophilized in reaction tubes equipped with a porous membrane (Greiner Bio-One, Germany). To prepare alginate inks, lyophilized alginate was dissolved in DPBS to a final concentration of 8% w/v. Gelatin (type A, gel strength 300) was obtained from Sigma-Aldrich, USA. Sterile gelatin solution was filtered and lyophilized as described above. To prepare alginate/gelatin bioinks, lyophilized alginate and gelatin were dissolved in DPBS to a final concentration of 2% w/v alginate and 10% w/v gelatin, respectively.

### 2.5 Bioinks and Bioprinting

NIH/3T3 and HUVECs (10 × 10^6^ cells/mL) were mixed into the inks using a direct displacement pipette. Bioinks were either cast in silicone moulds kindly provided by the University Hospital of Würzburg (dimensions: 6 mm diameter, 1 mm height) or printed using an extrusion printer (BioX, CELLINK, Sweden) through a 25 G nozzle (CELLINK, Sweden) at 25 kPa and 20 mm/s into 10 × 10 × 0.3 mm grids. The printhead was heated to 33 °C. After casting or printing, bioinks were cross-linked for 10 min at room temperature. The CELLINK bioink was cross-linked with a commercially available cross-linking agent (CELLINK, Sweden); alginate and alginate/gelatin bioinks were cross-linked with 2% w/v CaCl_2_ solution (Merck, Germany). Cross-linked bioinks were washed twice with DPBS containing Ca^2+^ (Life Technologies, USA) and subsequently incubated in cell culture media in a humidified incubator at 37 °C and 5% CO_2_ until analyzed. In experiments where both cell types were mixed, NIH/3T3 cells were labeled with 10 μM of CellTracker Green CMFDA (Life Technologies, USA) prior to harvesting.

### 2.6 Raman Microspectroscopy

Raman imaging was performed with a customized WITec alpha 300R Raman microscope (WITec GmbH, Germany), as described previously.^20^ Depth scans were performed over the full height of the bioinks (∼1 mm) at a resolution of 2 × 2 μm, a laser power of 50 mW, and an integration time of 0.1 s per pixel.

For imaging of single cells in the bioinks, an area of 20 × 20 μm was selected, and spectral maps were acquired at a resolution of 1 × 1 μm, a laser power of 50 mW, and an integration time of 0.1 s. For bioinks containing a mixture of NIH/3T3 fibroblasts and HUVECs, a larger area containing several cells (125 × 125 μm) was selected. Acquisition settings were the same as for single-cell imaging. At least 10 cells per batch were measured. Experiments were performed at least in triplicates. In addition, brightfield and fluorescence images were acquired of the selected mapping areas.

### 2.7 Data Analysis

Spectral image analysis was performed with the WITec Project FIVE 5.2 software (WITec GmbH, Germany). Data sets were preprocessed by cosmic ray removal, baseline correction, and normalization. True component analysis (TCA) was applied to generate images of the hyperspectral maps. Briefly, TCA is a non-negative matrix factorization-based algorithm that allows us to elaborate spectral signatures within the data set and creates intensity heatmaps for each identified spectral component that visualizes molecular distribution patterns. Further analysis to reduce dimensionality and classify the data was performed with the multivariate data analysis software, The unscrambler (Camo Analytics, Norway). Of each single-cell image, 50 spectra were randomly extracted and averaged to generate one representative average spectrum for each cell. These single-cell spectra were applied for principal component analysis (PCA) and linear discriminate analysis (LDA).

### 2.8 Statistical Methods

Statistical analysis was performed with Origin (OriginLab, USA). The Shapiro–Wilk test was applied to test for normality, and score values were compared by the non-paired *t*-test, one-way ANOVA, or Kruskal–Wallis for non-normally distributed data. *P*-values < 0.05 were considered statistically significant. Data are represented as mean ± SD. Experiments were performed at least in triplicates.

## 3 Results

### 3.1 Endothelial Cells and Fibroblasts Exhibit Individual Spectral Fingerprints

**Figure 1:**
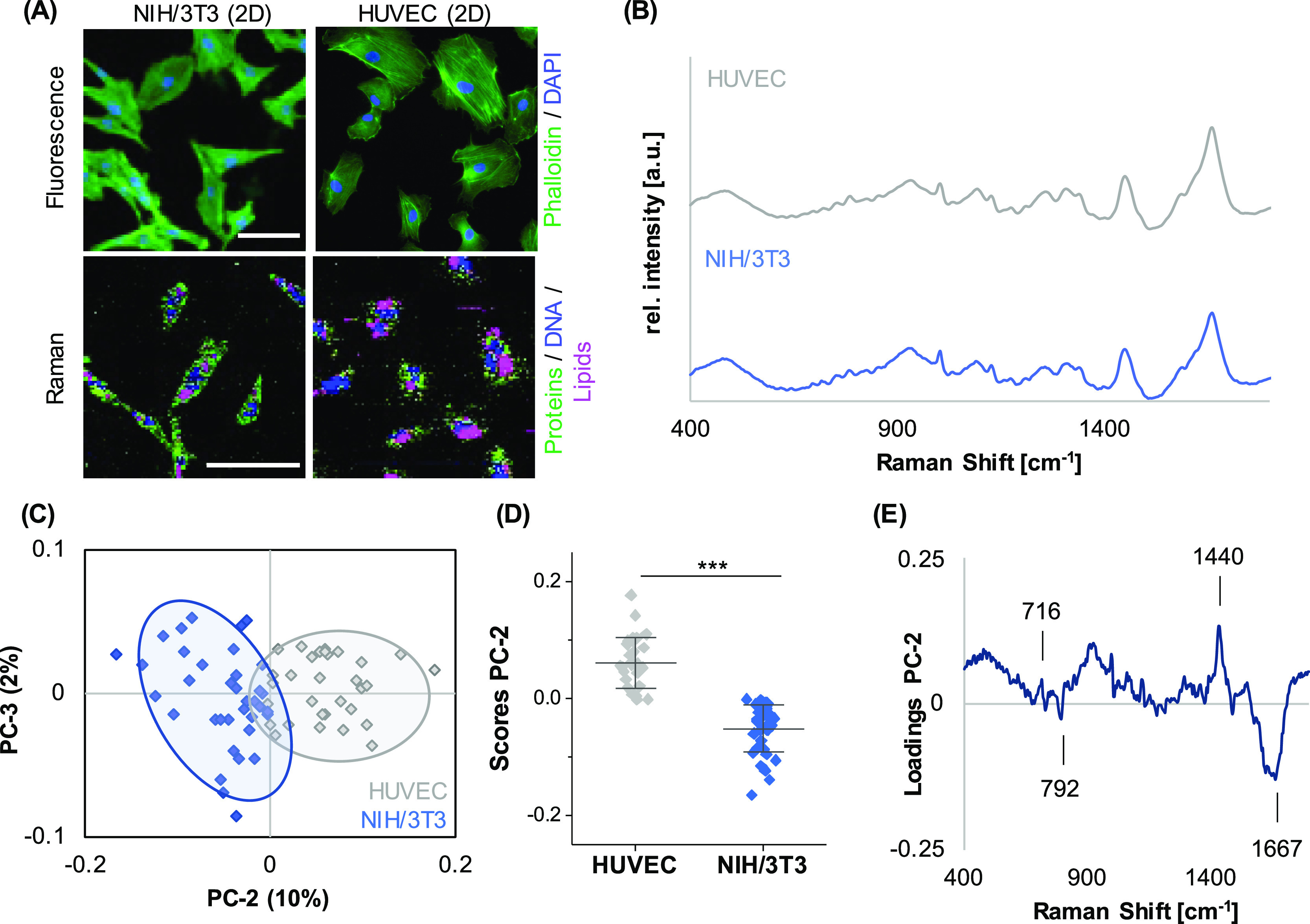
Different cell types in 2D culture can be distinguished based on their spectral fingerprint. (A) Upper panel: NIH/3T3 fibroblasts and HUVECs cultured on TCP were fixed and stained to visualize the actin cytoskeleton (green) and DNA (blue). Lower panel: Raman imaging to visualize the cellular components DNA (blue), lipids (pink), and proteins (green). Raman images revealed different proportions of the cellular components. In HUVECs, lipids appeared to be more abundant than in NIH/3T3 fibroblasts. (B) Representative single-cell average spectra for HUVECs (gray) and NIH/3T3 fibroblasts (blue). (C) PCA of single-cell average spectra demonstrated a clear clustering between the two cell types based on a difference in PC-2 score values. (D) Underlying spectral features linked to the observed separation are described by the PC-2 loading plot. (E) Non-paired *t*-test, *n* ≥ 30, *p ≤ 0.05. Scale bars equal 100 μm.

For initial parameter acquisition, HUVECs and human fibroblasts (NIH/3T3) were seeded in a 2D culture format on coated glass-bottom dishes, and Raman microspectroscopy was performed to elaborate cell-specific signatures. Raman mapping and subsequent TCA allowed us to localize lipids, proteins, and nuclei in both cell types, indicating a tendency toward a more pronounced lipid distribution in HUVECs when compared to NIH/3T3 cells (Figure 1A). Representative single-cell average spectra (Figure 1B) were extracted and further processed by PCA to investigate spectral shifts assigned to differences in the molecular composition (Figure 1C). PC-2 demonstrated a clear separation of both cell types (Figure 1D), with an accuracy of 94% determined by PCA–LDA. The underlying spectral differences can be explained by the corresponding PC-2 loading plot (Figure 1E) indicating increased lipid-related features (716 and 1440 cm^–1^) in HUVEC-derived spectra, whereas DNA (793 cm^–1^) and protein-related bands (1667 cm^–1^) dominated in the NIH/3T3 signatures. A comprehensive overview of all relevant peaks and their molecular assignments is provided in Table 1.

**Table 1:** Relevant Raman Peaks and Their Corresponding Molecular Assignments

Raman shift [cm^–1^]	molecular assignment
676	proteins
695	proteins
703	lipids
716	lipids
750	cytochrome c
792	DNA
1003	phenylalanine
1084	proteins
1130	cytochrome c
1305	lipids
1332	DNA
1342	CH_3_ deformation, DNA methylation
1435–1444	CH deformation, lipids
1452	CH deformation
1584	cytochrome c
1655–1660	lipids
1667	amide I, proteins

### 3.2 Biomaterial Ink Composition Influences the Optical Penetration Depth

**Figure 2:**
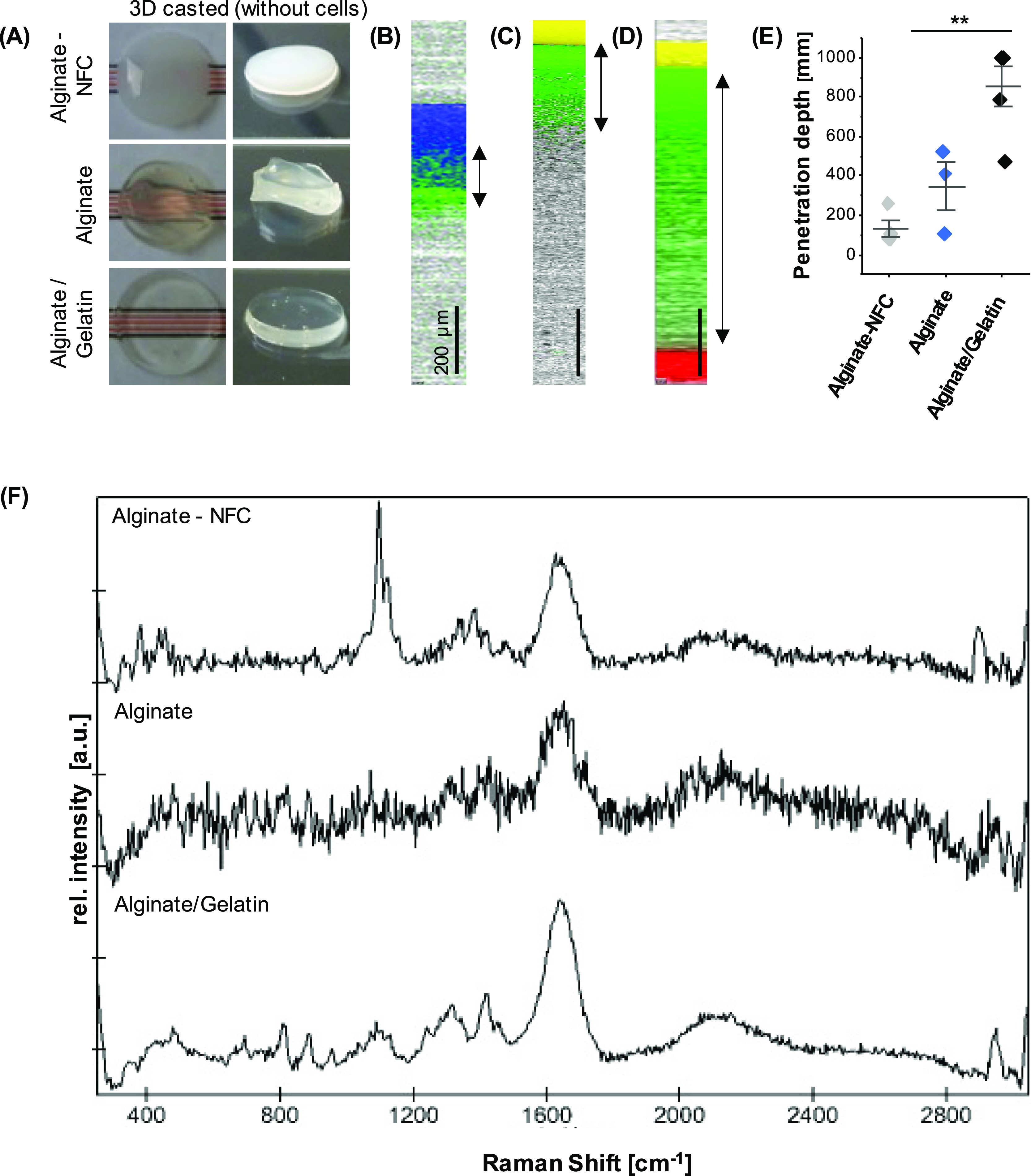
Penetration depth of Raman spectroscopy in different biomaterial inks without cells. (A) Inks were cast into gels of approx. 1 mm in height. (B–D) Raman images were acquired in the *X*–*Z*-direction over the full depth of alginate-NFC (B), alginate (C), and alginate/gelatin (D) inks. The ink (green), glass (yellow), water (blue), and the basic coating (red) were localized based on their different spectral signatures. Scale bars equal 200 μm.(E) Penetration depths were determined in the Raman images. Mean and SD of three independent samples, Kruskal–Wallis test, *p ≤ 0.05. (F) Background spectra of inks.

Three different biomaterial inks were analyzed to determine the optical behavior and penetration depth of Raman scattering. First, inks of 1 mm height and without cells were generated from alginate-NFC (CELLINK), alginate, and alginate/gelatin. Macroscopic images of the inks showed different optical densities (Figure 2A). Although the alginate-NFC ink resulted in white, opaque gels, the alginate/gelatin ink was transparent. The inks were transferred to coated cell culture dishes, and depth scans were performed to identify the penetration characteristics in every ink (Figure 2B–E). Alginate-NFC showed the poorest penetration properties for Raman scattering; a signal could no longer be detected after 148 (±50) μm. In alginate, signal detection was possible up to a depth of 346 (±125) μm, and alginate/gelatin inks provided the best optical properties. The Raman signal was traceable throughout the full depth of the ink. Each ink material provided a specific spectral signature (Figure 2F).

### 3.3 Raman Imaging Allows Discrimination and Localization of Subcellular Components in a Bioink Environment

**Figure 3:**
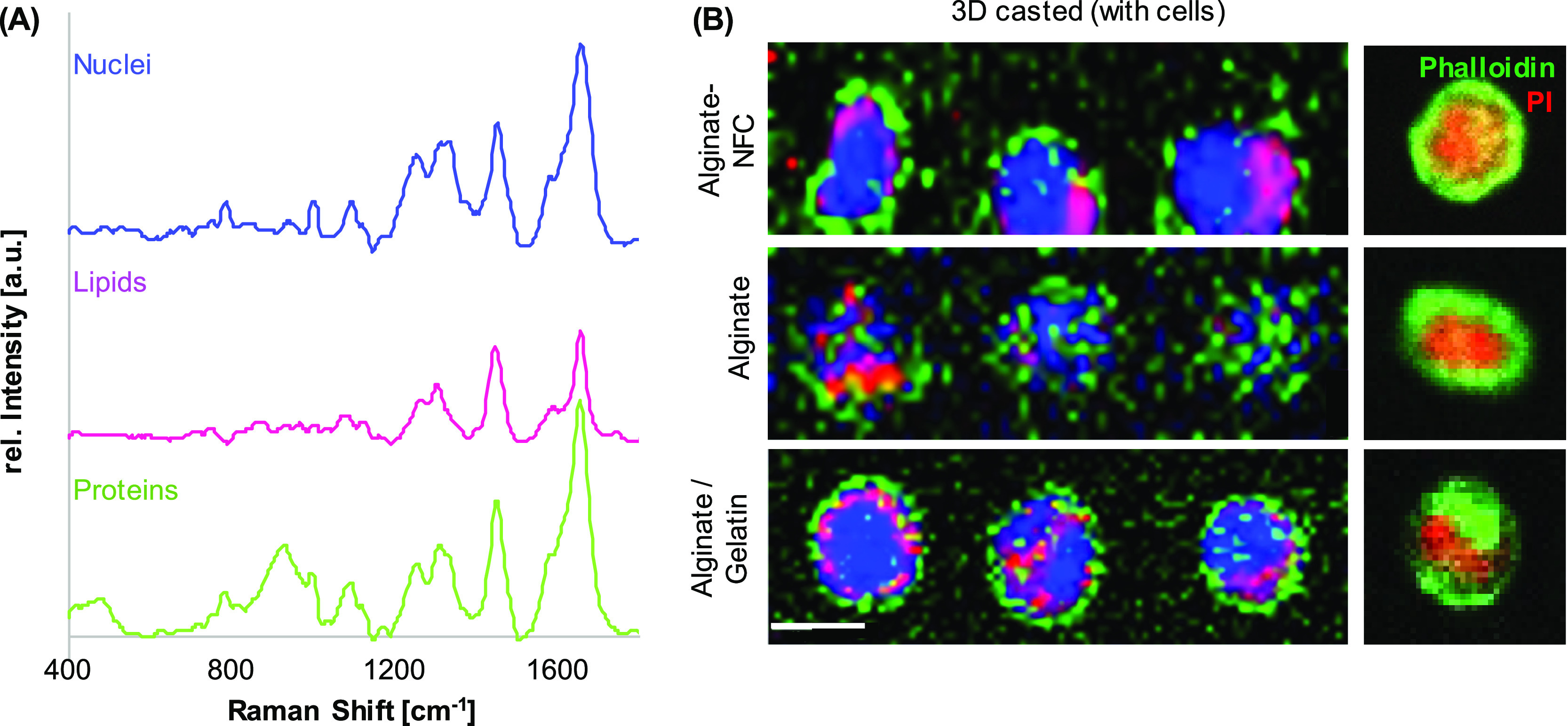
Raman imaging of NIH/3T3 fibroblasts in alginate, alginate-NFC, and alginate/gelatin bioinks. TCA identified three spectral signatures assigned to DNA (blue), lipids (pink), and proteins (green) (A) and allowed us to visualize their distribution within the cells (B). Actin cytoskeleton and nuclei of NIH/3T3 fibroblasts were labeled with phalloidin oregon green (green) and propidium iodide (red), respectively. Scale bar equals 10 μm.

The next step aims to transfer the acquisition and analysis parameters established for 2D cultured cells of 3D composites—not only to localize cells within the bioink but also to assess their phenotype and spatially resolve their subcellular composition in a 3D environment. Thus, NIH/3T3 fibroblasts were embedded in alginate, alginate/NFC (CELLINK), or alginate/gelatin. After 24 h of incubation, single-cell spectral maps were acquired for cells in the upper bioink layer (∼300 μm) and analyzed by TCA. Three major spectral components were identified in all bioinks (Figure 3A). Spectral assignments of these components can be linked to DNA, lipids, and proteins. Intensity heatmaps allowed us to demonstrate the distribution of the cellular components in contrast to the bioink background (Figure 3B). The alginate/gelatin bioink showed the best imaging properties with the least signal-to-noise ratio and background interference, which were especially exhibited in the alginate ink without additives.

### 3.4 Cell Types Can Be Distinguished in Cast and Printed Bioinks

**Figure 4:**
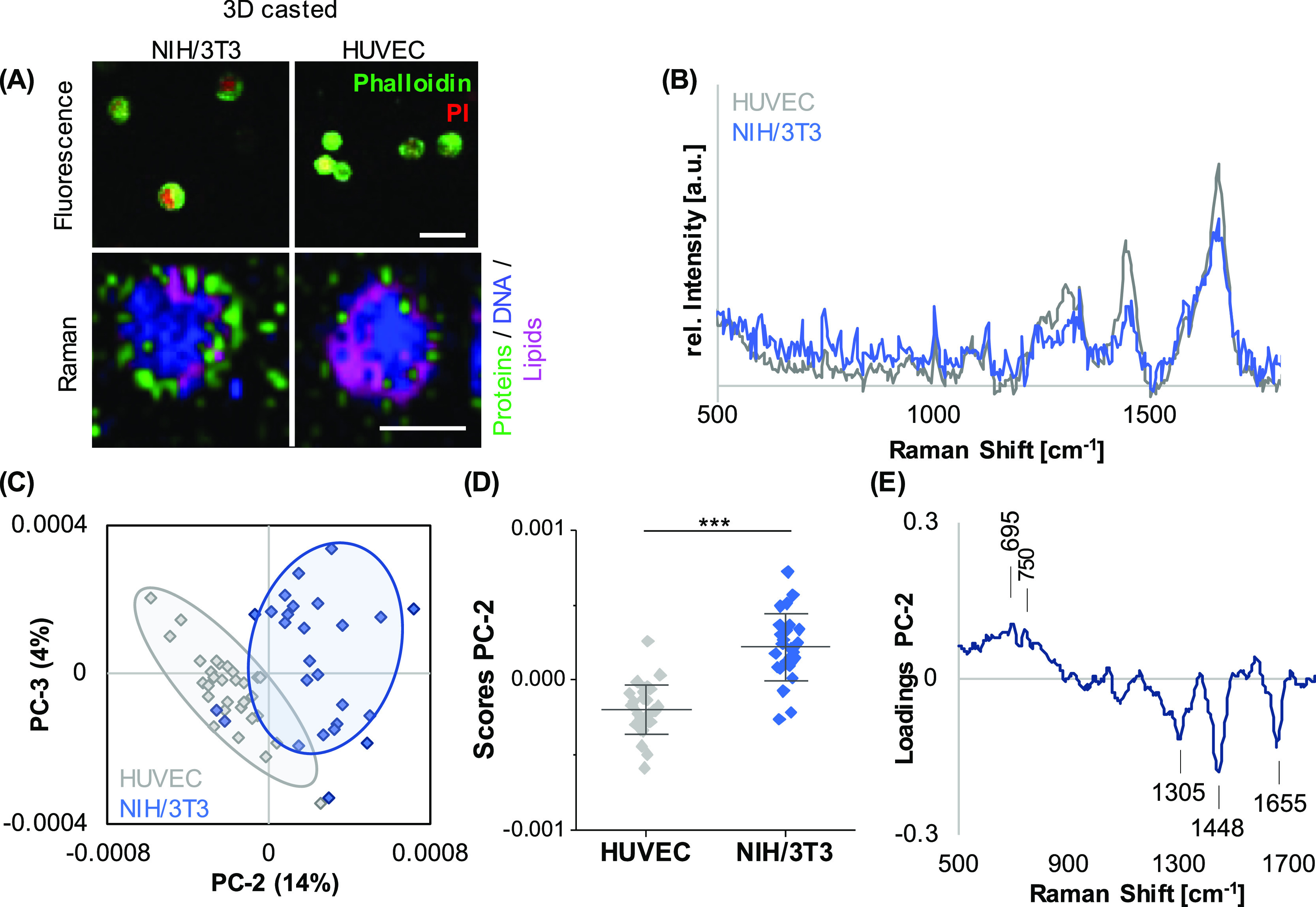
Raman imaging of different cell types in alginate/gelatin bioink. (A) NIH/3T3 fibroblasts or HUVECs were separately embedded in alginate/gelatin bioink and analyzed in situ by fluorescence microscopy, Raman imaging, and TCA. Upper panel: the actin cytoskeleton of NIH/3T3 fibroblasts and HUVECs was stained with phalloidin oregon green (green) and nuclei were labeled with propidium iodide (red). Cells were imaged by confocal microscopy. Scale bar equals 25 μm. Lower panel: Raman imaging of NIH/3T3 fibroblasts and HUVECs to visualize cellular component DNA (blue), lipids (pink), and proteins (green). Scale bar equals 10 μm. (B) Representative average spectra for HUVECs and NIH/3T3 fibroblasts. (C) Single-cell average spectra were compared by PCA. PC-2 indicated clustering of individual celltypes (C,D). Spectral differences are described in the loading plot (E). Non-paired *t*-test, *n* ≥ 30, *p ≤ 0.05.

Alginate/gelatin-based bioinks were further investigated in subsequent experiments due to their advantageous optical properties. In addition to NIH/3T3 fibroblasts, HUVECs were embedded in separate alginate/gelatin bioinks. Raman images were acquired for both cell types in order to test the sensitivity of Raman microspectroscopy to distinguish different cells in a bioink environment (Figure 4A). Single-cell average spectra were extracted (Figure 4B) and analyzed by PCA. The PC score plot (Figure 4C) demonstrated a significant separation of spectral information originating from both cell types (Figure 4D). According to the corresponding PC-2 loading plot (Figure 4E), similar peaks were identified as major spectral features, as already shown for the 2D cultured HUVECs and fibroblasts. HUVECs demonstrated a major influence of lipid-related bands on their fingerprint spectra (1305, 1440, and 1655 cm^–1^). The PCA–LDA classification confirmed a high discriminational power of the model with an accuracy of 98%.

**Figure 5:**
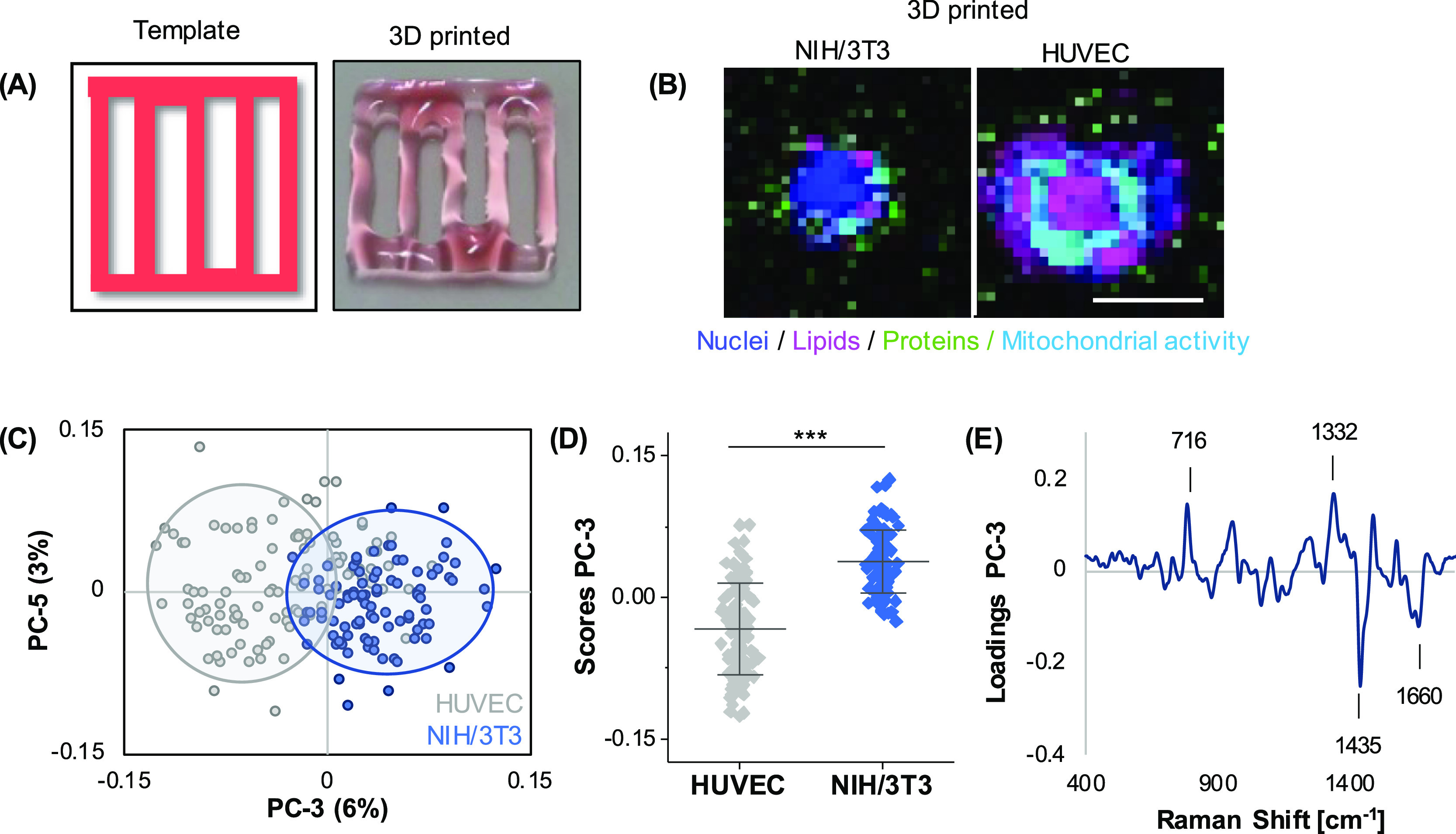
Different cell types in printed alginate/gelatin bioinks can be distinguished based on their spectral fingerprints. (A) Scheme and representative macroscopic image of a printed 10 × 10 × 0.3 mm grid. 2.5 mg/mL of cochenille red was added to alginate/gelatin bioink for better visualization. (B) Raman imaging of printed objects of alginate/gelatin bioinks containing NIH/3T3 fibroblasts or HUVECs was performed to visualize cellular component DNA (blue), lipids (pink), and proteins (green). In addition to previous TCAs, an additional spectral component was identified that could be assigned to mitochondrial activity (light blue). (C,D) PCA analysis of single-cell average spectra reveals a clear separation of both cell types in PC-3. (E) Correlating PC-3 loading plot. Non-paired *t*-test, *n* ≥ 60, *p < 0.05. Scale bar equals 10 μm.

In the next step, the established method was transferred from cast bioinks to printed bioinks. Grid structures were generated with a pneumatic extrusion printer (Figure 5A). Raman image acquisition and TCA were performed on HUVECs and fibroblasts printed separately in alginate/gelatin bioinks, respectively. Interestingly, an additional major spectral component was identified in both cells—but predominantly in HUVECs—which could be assigned to mitochondrial features (Figure 5B). Bioinks with the different cell types were distinguished by PCA (Figure 5C), with a significant shift demonstrated by PC-3 (Figure 5D) and an accuracy of 96%. Major spectral differences were linked to membrane lipids (716 cm^–1^) and DNA (1332 cm^–1^) in fibroblasts and lipids (1435 and 1660 cm^–1^) in HUVECs (Figure 5E).

### 3.5 Culture Format and Cell Type-Specific Signatures Can Be Discriminated by Multivariate Analysis

**Figure 6:**
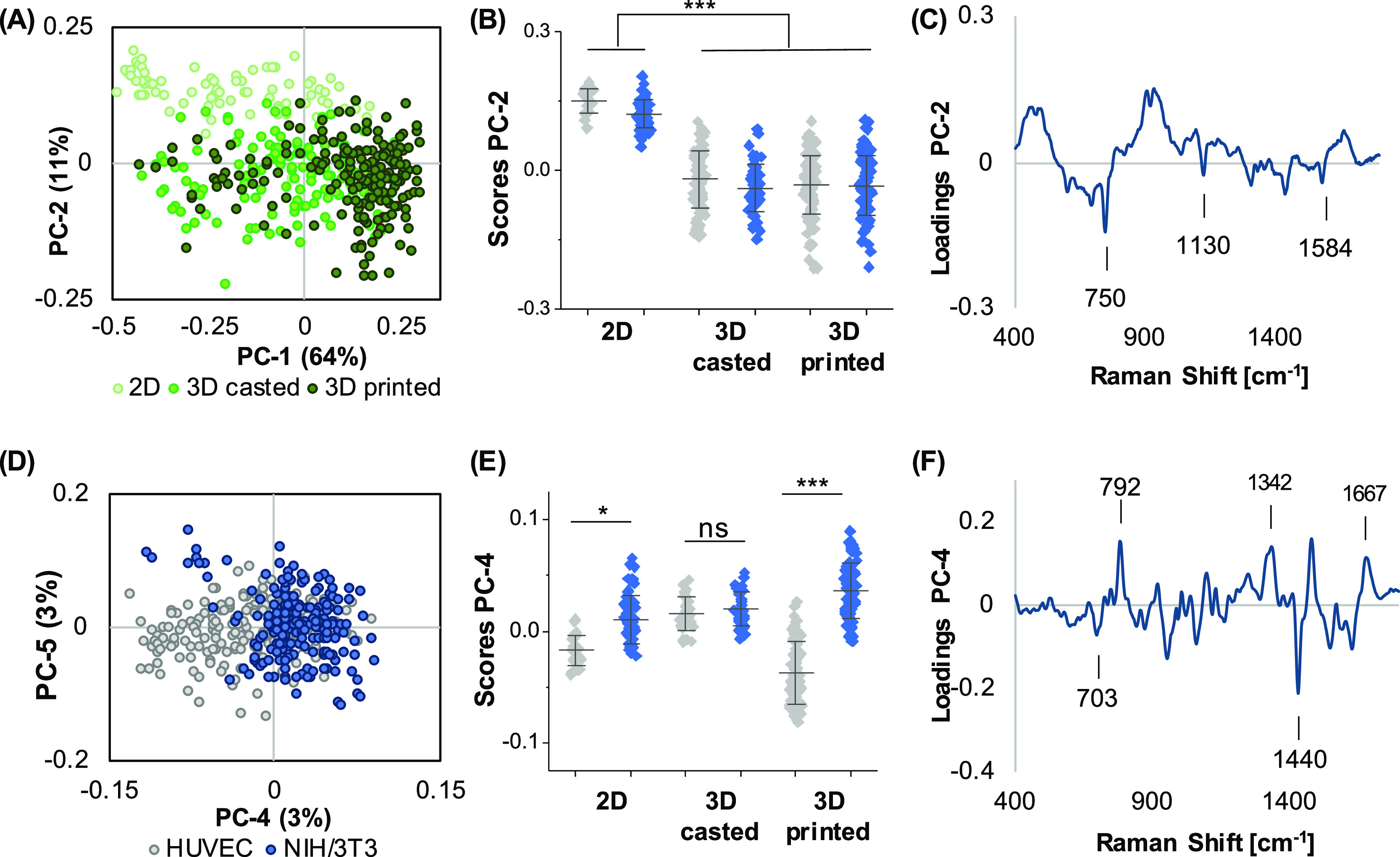
Cell discrimination in different culture formats. PCA of the full data set including both cell types in 2D and 3D (cast and printed) conditions was performed. (A,B) PC-1/PC-2 scores plot reveals a clustering between 2D and 3D printed cells in PC-2, differing significantly from 2D, which is correlated to the spectral signatures demonstrated in the PC-2 loading (C). PC-4/PC-5 score plot (D) still enables differentiation of both cell types by PC-4. (E) One-way ANOVA of PC-4 score values for each origin did not allow for cell distinction within the 3D cast group but within the 2D and 3D printed groups. Spectral features for the cell type discrimination are described in the loading plot (F). *n* ≥ 30, *p ≤ 0.05.

In order to evaluate a potential influence on the cellular signature by the culture format (2D, cast, or printed), an additional PCA was performed including all previously measured HUVECs and NIH/3T3 cells. Depending on the different PCs, several effects could be demonstrated by PCA. PC-1 versus PC-2 score plot (Figure 6A) showed a clear separation between 2D and 3D cultured cells in PC-2 (Figure 6B). Spectral features explaining these observations were assigned to increased bands at 750, 1130, and 1582 cm^–1^ in the bioinks (Figure 6C). These bands correlate with cytochrome c, which is linked to mitochondrial activity. Despite the major influence of the culture format, it was still possible to discriminate both cell types throughout different casting methods. Clustering in the PC-4 versus PC-5 score plot was shown between HUVECs and fibroblasts (Figure 6D). This separation was based on previously detected features from previous PCAs (Section 3.4), which turned out to be robust indicators for cellular origin. Solely 3D cast cells did not show a clear separation in the overall comparison (Figure 6E). HUVECs predominantly exhibited the CH_3_ deformation band of lipids at 1440 cm^–1^, whereas NIH/3T3 cells were characterized and classified by an increased DNA peak (793 cm^–1^) (Figure 6F).

**Figure 7:**
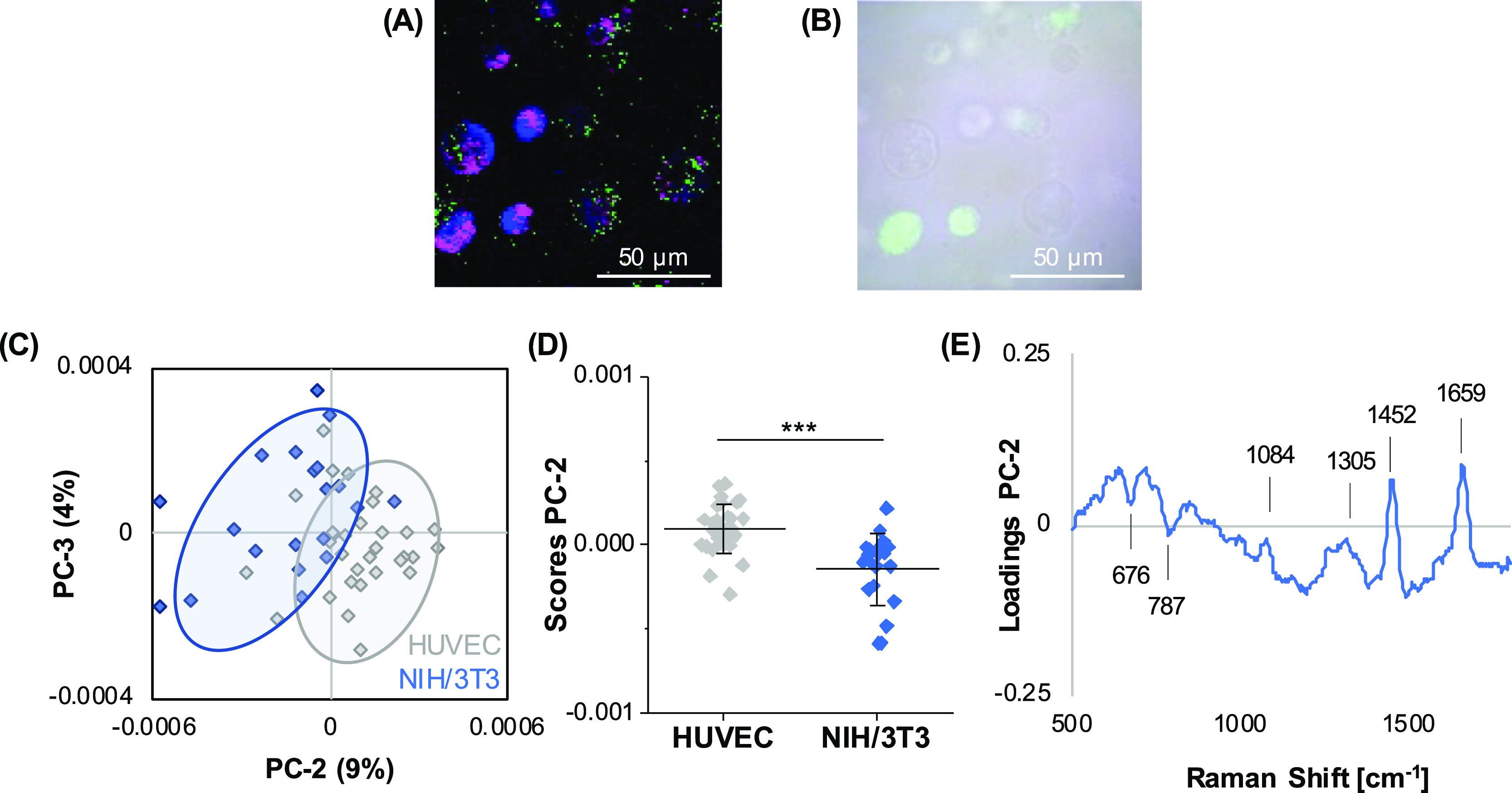
Raman analysis of a mixture of different cell types in cast alginate/gelatin bioinks. Single-cell average spectra were extracted from hyperspectral maps generated of bioinks containing a mixture of HUVECs (unlabeled) and NIH/3T3 cells (CellTracker Green positive) (A). Fluorescence/brightfield overlay images served as the control (B). PCA score plot (C) and comparison of PC score values (D) revealed a clustering within both cell types in PC-2. Spectral differences between the groups are indicated in the loading plot (E). Non-paired *t*-test, *n* > 30, *p ≤ 0.05.

Finally, HUVECs and NIH/3T3 cells were combined in the same alginate/gelatin bioink and assessed by Raman imaging (Figure 7A). Fluorescence labeling of NIH/3T3 cells with CellTracker Green served as a control to validate the PCA classification (Figure 7B). Again, both cell types could be distinguished by PCA analysis with the most relevant peaks for discrimination assigned to lipids (1305, 1452, and 1659 cm^–1^) in HUVECs as well as DNA (792 cm^–1^) and proteins (676 cm^–1^) in fibroblasts (Figure 7C–E). The overall accuracy determined by PCA-LDA was 85%, with a correct classification of 100% for HUVECs and 87% for NIH/3T3 cells.

## 4 Discussion

Standard methods for the characterization and discrimination of distinct cell types in bioprinted objects often include invasive end point measurements such as immunofluorescent staining of cell type-specific marker proteins.^21,22^ All commonly used fluorescence microscopy-based methods to distinguish cell types are limited by the optical properties of the bioinks, destructive sample preparation such as fixation and sectioning, and the permeability of dyes or antibody staining. Non-invasive strategies to discriminate different cells are stable transfection of cells with fluorescent proteins^23^ and pre-labeling of cells with fluorescent tracking dyes^24^ prior to mixing them into the bioink. Furthermore, fluorescent beads can be added in the formulation of bioinks. When different cell types are encapsulated in different bioinks containing fluorescent beads of a different color, the patterning of the printed inks can be visualized after printing.^22^ Another approach is to label the biomaterial in the ink formulation itself.^25^ Since the cells are not labeled themselves, these methods do not allow the observation of their morphology nor their interaction with the biomaterial and with each other. Thus, we established and validated Raman microspectroscopy combined with multivariate data analysis as a non-invasive tool for bioink characterization, the identification of cell types, as well as for the localization and discrimination of subcellular structures.

First, Raman microspectroscopy was performed on 2D cultures of the model cell types, endothelial cells (HUVECs), and fibroblasts (NIH/3T3) used in this study to elaborate cell-specific signatures. The sensitivity of Raman spectroscopy to discriminate fibroblasts and epithelial cells has already been reported by Ichimura et al., who were able to discriminate NIH3T3 fibroblasts and EPH4 epithelial cells based on the nucleus signature.^26^ PCA of single-cell average spectra revealed increased lipid-related features in HUVECs when compared to fibroblasts, whereas in NIH/3T3 cells, protein-related bands dominated.

For 3D bioprinting, cells are encapsulated in bioinks which have to be printable and must also maintain cell viability before, during, and after the printing process. To achieve all these properties, often a mix of two or more biomaterials is used in extrusion bioprinting approaches.^27^ Alginate-based bioinks have been used in a variety of applications with a number of different cell types and have been mixed with biomaterials such as gelatin,^4^ NFC,^12,14^ matrigel,^28^ and collagen.^29^ Raman microspectroscopy provides information on the molecular composition of a sample and has been broadly used to characterize biopolymers such as collagen, silk-fibroin, or biopolymer composites,^30−32^ which are also applicable for 3D bioprinting. The selection of a certain biopolymer or biopolymer composite does not only influence printability, mechanical properties, and cell viability^5,27^ but equally has an impact on optical properties, which was demonstrated in-depth scans of different bioink formulations and might hamper accessibility for analysis by microscopic methods. In our study, signal penetration was enabled in all tested polymers but to significantly different degrees ranging from below 200 μm in alginate-NFC polymers to the full thickness of 1 mm in alginate–gelatin composites. NFC particles show fibril-like morphology with microns in length and nanometers in width.^33^ Although the cellulose nanofibers are very thin (2–10 nm), they scatter light due to their assembly into fiber bundles, thus limiting optical detection.^34^ Deeper signal penetration in the alginate–gelatin hydrogel compared to the pure alginate hydrogel might occur due to the lower alginate concentration in the composite. Besides cell-free biomaterial inks, bioinks with NIH/3T3 fibroblasts were generated to analyze the z-scan imaging properties in bioinks with cells. Depth scans were able to visualize cells to a depth of 1000 μm. However, cells in the bioink can lead to a loss of signal intensity in deeper layers, decreasing the overall penetration depth (Supporting Information Figure S2). To improve the penetration depth in thicker samples with a high cell density, a near-infrared laser instead of a green laser might enable even deeper tissue penetration.^35^

In addition to material characterization, Raman spectroscopy and imaging are constantly gaining significance in the biomedical field, especially for the identification and analysis of cellular features such as differentiation, disease state, or cell death.^17,36,37^ Most commonly, cells are analyzed in a 2D or tissue environment. Baldock et al. demonstrated an approach for single-cell Raman imaging in 3D micro-engineered cell scaffolds,^38^ but up to date, little has been reported on the suitability of Raman microspectroscopy as an in situ tool to monitor cells in 3D cast or bioprinted samples. A study by Othman et al. applied Raman spectroscopy on microtissues retrieved from HeLa spheroids printed on alginate–gelatin bioink scaffolds. However, microtissues were separated from the bioink construct before measurements, which were then also limited to single spectrum acquisition.^39^ Therefore, in our study, we focused on in situ measurements and spatial resolutions of subcellular features.

Subsequent to optical penetration depth characteristics, bioinks were evaluated according to their sensitivity to detect cellular features on a biopolymer background. Despite a protein signal arising from the bioink substrates, cellular proteins and other subcellular structures could be clearly discriminated from biopolymers. Accordingly, Raman imaging does not only enable us to localize and identify cells in a 3D printed microenvironment but also allows us to trace subcellular changes in the lipidome, protein composition, metabolic activity, or epigenetics, as it has already been demonstrated for 2D and 3D cell culture.^40−42^

Moreover, spectral signatures of two exemplary cell types—fibroblasts and endothelial cells—were acquired and shown to be highly specific to consistently distinguish the two different cell types. Single-cell Raman fingerprints have proven their sensitivity to discriminate various cell types or subtypes in previous studies,^18,43,44^ and our study demonstrates robust discrimination of HUVECs and NIH/3T3 cells throughout different 2D and 3D printed culture formats. Data acquisition parameters and analysis could be transferred between 2D and 3D samples, and spectral signatures expressed the same features distinguishing the two cell types at similar accuracies in 2D and 3D cultures. The spectral sum fingerprint of endothelial cells was dominated by lipid-related features, whereas fibroblasts expressed strong protein and DNA bands. These observations correlate to the physiological function of both cell types as fibroblasts are highly synthetic and proliferative and produce extracellular matrix proteins,^45^ whereas in an endothelial cell membrane, integrity and signal transduction are essential.^46^ Even though the discrimination between the different cell types was enabled in all experimental setups presented in this work, the co-culture bioink containing both cell types showed the least accurate separation with 85% (validated by PCA–LDA). These observations might be a consequence of culture conditions (50% HUVEC medium; 50% fibroblast medium) or reciprocal stimulation, for example, by the vascular endothelial growth factor, inducing alterations in proliferation or the phenotype.^47−49^ Due to the sensitivity of Raman spectroscopy, these changes can be reflected in spectral signatures accordingly, when compared to cells in mono culture.

Besides the discrimination based on the cell type, multivariate data analysis allowed us to determine an additional feature separating the cells according to their culture format. Significant spectral shifts could be observed in the signatures of 2D and 3D cultured cells, whereas 3D casting resulted in similar score values when compared to 3D printing. The underlying differences in molecular compositions between 2D and 3D cultured cells mainly referred to an increased mitochondrial activity in 3D formats and could be a result of the applied shear forces during extrusion-based bioprinting and previous sample preparation. Compared to other cell types, fibroblasts and endothelial cells are relatively insensitive to effects on their viability upon shear forces in bioprinting.^50^ However, especially in endothelial cells, shear stress regulates reactive oxygen species production^51^ and influences the lipid composition,^52^ which correlates to the observed spectral changes between 2D and 3D cultured cells. The current Raman study not only recapitulates cellular phenotypes 24 h after seeding, casting, or printing in 3D but also shows the potential for follow-up experiments to monitor cell maturation over a longer timeline and observe cellular changes such as phenotypic, metabolic, or maturation processes. Furthermore, a major limitation of spontaneous Raman imaging of larger objects at a high spatial resolution is represented in the acquisition speed. Our data encourage us to use a representative number of single-cell scans to perform cellular phenotyping at high sensitivity. However, image generation of a whole bioprinted scaffold at the presented spatial resolution would require larger spectral scans. To achieve this in a feasible timeframe, signal enhancing techniques such as coherent anti-stokes Raman scattering (CARS) or stimulated Raman scattering could be applied based on the identified spectral bands relevant for the cellular discrimination.

## 5 Conclusions

The presented results show the potential of molecular-sensitive Raman imaging as a real-time quality control tool for biofabrication, which can allow us to monitor both the impact of the bioprinting process as well as the phenotype and functionality of the integrated cells in a time-resolved manner. In the future, applications of bioprinted scaffolds for both physiological in vitro models as well as tissue or organ replacements aim to be implemented as personalized, on-demand, and point-of-care solutions and will require on-line quality control for safety assessment—similar to the quality by the design approach pursued in pharmaceutical production. Moreover, with sophisticated machine learning-based data analysis tools, automatization and real-time monitoring with a direct feedback loop to the printing process could be facilitated. Thus, our results provide the first proof-of-principle of Raman imaging as the in situ tool to monitor bioprinted products and their maturation and highly encourage us to implement and further evolve this technique for quality assessment in biofabrication.

## Supporting Information Available

Prevention of floating of printed objects from the cell culture substrate by coating and penetration depth in cast bioinks including cells (PDF)
