# Supplementary material for: Non-Invasive Three-Dimensional Cell Analysis in Bioinks by Raman Imaging
Source: ACS Appl Mater Interfaces. 2022 Jul 1;14(27):30455–65. doi: 10.1021/acsami.1c24463 (PMC9284518; doi:10.1021/acsami.1c24463)
Supplement: Supplementary file 1 — am1c24463_si_001.pdf [file am1c24463_si_001.pdf]

Supporting Information

Non-invasive three-dimensional cell analysis in bioinks by Raman imaging

Julia Marzi ‡ [1, 2, 3], Ellena Fuhrmann ‡ [1], Eva Brauchle [1, 2, 3], Verena Singer [1], Jessica Pfannstiel [1], Isabelle Schmidt [1], Hanna Hartmann * [1]

‡ authors contributed equally

[1] NMI Natural and Medical Sciences Institute at the University of Tübingen, Reutlingen, Germany

[2] Institute of Biomedical Engineering, Department for Medical Technologies & Regenerative Medicine, Eberhard Karls University Tübingen, Germany

[3] Cluster of Excellence iFIT (EXC 2180) "Image-Guided and Functionally Instructed Tumor Therapies", University of Tübingen, Germany

**Corresponding author: Dr. Hanna Hartmann, NMI Natural and Medical Sciences Institute at the University of Tuebingen, Markwiesenstraße 55, 72770 Reutlingen, Germany, hanna.hartmann@nmi.de, +49712151530872*

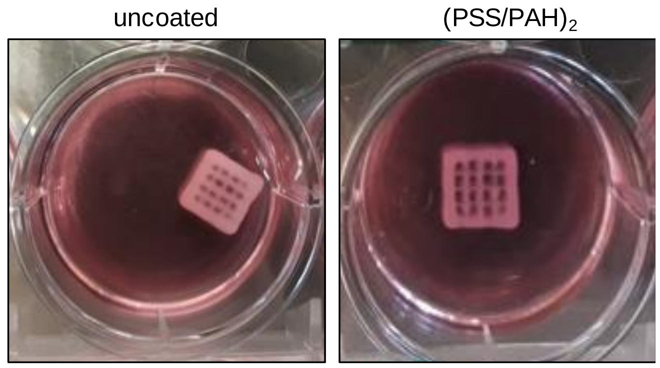

	uncoated	$(PSS/PAH)_2$
Crosslinked	floating	adhesion
Not crosslinked	floating	adhesion
Contact angle [°]	60 ± 1	41 ± 4

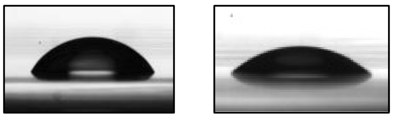

Figure S1: Coating prevents floating of printed objects from cell culture substrate. Cell culture dishes were coated with PEI and two bilayers of PSS/PAH. Successful coating was verified by a decreased contact angle. Grids were printed on coated and uncoated substrates and floating of crosslinked and uncrosslinked objects was probed by the addition of cell culture medium.

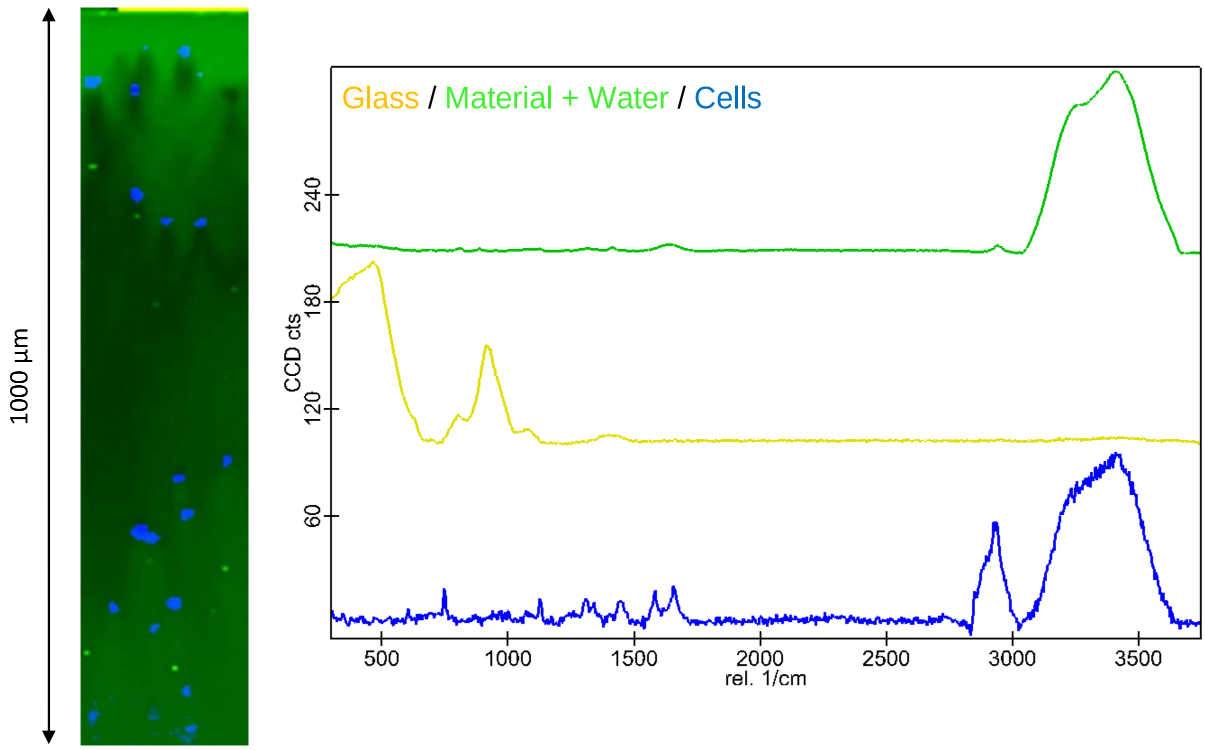

Figure S2: Penetration depth in casted bioinks including cells. Raman depth scans in casted alginate/gelatin bioinks detect cells to a depth of 1,000 µm. Decreases in signal intensities are visible, especially for the material signal below cells. TCA color coding: alginate/gelatin (green), glass coverslip (yellow), nuclei (blue). Image dimensions 1000 x 200 µm (depth x width).
